# Preoperative Indicators of the Effectiveness of Surgical Release in Patients with de Quervain Disease: A Prospective Cohort Study

**DOI:** 10.1097/PRS.0000000000010445

**Published:** 2023-11-23

**Authors:** Romy Bosman, C. A. Hundepool, Mark J. W. van der Oest, Liron S. Duraku, J. S. Souer, Ruud W. Selles, J. Michiel Zuidam

**Affiliations:** Rotterdam and Amsterdam, the Netherlands; From the Departments of 1 Plastic, Reconstructive and Hand Surgery; 2 Rehabilitation Medicine, Erasmus MC, University Medical Center Rotterdam; 3 Department of Plastic, Reconstructive, and Hand Surgery, Amsterdam UMC; 4 Hand and Wrist Center, Xpert Clinic.

## Abstract

**Background:**

A significant proportion of patients report persistent pain after surgical release for de Quervain disease (DQ). This study aimed to investigate the effectiveness of a surgical release for DQ and to identify the preoperative factors associated with pain after a surgical release for DQ.

**Methods:**

This prospective cohort study included 707 patients who underwent surgical release and completed a visual analogue scale questionnaire (VAS; range 0 to 100). We used a paired *t* test to analyze the effectiveness of the surgical release on pain at 3 months postoperatively compared with the preoperative measure. A hierarchical multivariable linear regression model was created to investigate the contribution of patient-related and disease-related characteristics to postoperative pain.

**Results:**

All VAS domains showed improvement after surgical release. On average, the mean VAS pain decreased by 44 points (95% CI, 42, 46). Smoking (B = 6.37; *P* < 0.01), younger age (B = −0.35; *P* < 0.01), longer duration of complaints (B = 0.13; *P* < 0.01), concomitant surgery (B = 14.40; *P* < 0.01), and higher VAS pain scores at intake (B = 0.15; *P* < 0.01) were associated with worse VAS pain scores postoperatively. Together, the variables explained 11% of the variance in mean VAS pain score at 3 months follow-up.

**Conclusions:**

This study confirms that surgical treatment for DQ significantly reduces patient-reported pain. Smoking, younger age, concomitant surgery, duration of complaints, and higher VAS pain scores at intake are associated with worse patient-reported pain 3 months after surgical release. However, the small effects suggest that these factors should not be considered the only important factors.

**CLINICAL QUESTION/LEVEL OF EVIDENCE:**

Risk, III.

De Quervain disease (DQ) is a common upper limb disorder for which surgery provides relief of pain and an increase in function in most patients. DQ has a prevalence of 1.3% among women and 0.5% among men in the general population.^1^ It is primarily treated conservatively, with a splint or corticosteroid injections; however, surgery is often required. The primary goal of surgical release is to resolve pain and improve function, but a significant proportion of patients maintain symptoms postoperatively.^2–5^ Patients report an average decrease of 5 to 6 points on the visual analogue scale (VAS) (range, 0 to 10) for pain after open surgical release for DQ.^6–8^ Despite this reported decrease in pain, the percentage of patients still reporting pain following surgery ranges from 5% to 34%.^9,10^

Studies have shown that a negative mindset is associated with worse outcomes, but literature regarding the effect of clinical risk factors on functional outcomes of surgery for DQ remains scarce. Literature shows that psychologic factors (ie, lower patient expectations before surgery, negative illness perception, greater emotional distress) are negatively associated with patient-reported outcomes after surgical release for DQ.^11–13^ Less is known about clinical risk factors. Bryant et al.^14^ reported that diabetes and smoking were independent risk factors for complications after surgery for DQ. However, they used the complication risk as an outcome measure to identify prognostic factors. Furthermore, the authors did not assess other clinical factors, such as symptom duration, previous conservative treatment, or more general factors, such as sex, age, or medical history of rheumatoid arthritis, lupus, or hypothyroidism.^15^ These factors are of interest to investigate because some are associated with an increased risk of diagnosis or have already been shown to affect outcomes after surgery in other hand conditions, such as trigger finger.^14,16^ Because individual outcomes vary significantly after surgical release for DQ, it would be valuable to provide insight into whether clinical prognostic factors affect the outcome of surgical treatment for DQ.

This study aimed to assess the effectiveness of surgical release and determine the preoperative clinical factors associated with pain after surgical release for DQ in a large study population. Obtaining knowledge of these factors and their effect on postoperative pain could help improve preoperative consultations, manage patient expectations, and improve patient-reported outcomes postoperatively.

## PATIENTS AND METHODS

### Study Design

This cohort study used data from a longitudinally maintained electronic database of patients at Xpert Clinics Hand and Wrist care, comprising 25 outpatient clinics for hand surgery and therapy in the Netherlands. More detailed information about design, development, and implementation of this routine outcome measurement cohort has been provided in the literature.^17^ All patients provided digital permission to use their data anonymously for scientific research. If patients agreed, they received internet-based questionnaires at baseline and follow-up after intervention. Ethical approval for this study was obtained from the medical ethics review committee of the Erasmus Medical Centre (METC-2018-1088).

### Participants

All patients older than 18 with a clinical diagnosis of DQ in whom conservative therapy failed and who elected to undergo surgical treatment between December of 2011 and September of 2019 were included. Patients were excluded if they previously underwent surgical release for DQ; they had undergone surgical treatment of the ipsilateral or contralateral hand within 3 months before or after surgical release for DQ; there were missing baseline characteristics; it was unknown whether they received steroid injections preoperatively; or they did not complete the VAS at intake or 3-month follow-up.

### Treatment

All patients underwent a surgical release for DQ performed by hand surgeons certified by the Federation of European Societies for Surgery of the Hand. Most patients received local anesthesia. Incisions were made according to the surgeon’s preference (eg, transverse, longitudinal, lazy-S, or oblique), but most commonly, a transverse incision was used. The extensor retinaculum was incised longitudinally on the compartment’s dorsal side. When needed, additional subcompartments were also released. All patients received standard postoperative care, consisting of 3 days of pressure garments. Hand therapy was offered to all patients. After 3 to 5 days, patients had their first appointment with their hand therapist. In this appointment, tendon gliding, abduction and adduction, and ulnar and radial deviation exercises were practiced. The second appointment took place after 10 to 14 days when sutures were removed, and scar massage started. Three months postoperatively, patients were evaluated by surgeons to assess treatment outcomes.

### Variables

Sociodemographic data were collected prospectively before surgery and classified into three categories: patient characteristics, including age, sex, body mass index (BMI), type of work and workload, smoking, hand dominance, and medical history; disease-specific characteristics, including duration of complaints (determined as the time reported by patients they first perceived symptoms as recorded by the hand therapist), operation side, and preoperative steroid injections; and preoperative hand function and pain. To assess pain as a primary outcome measure, patients filled in a VAS for pain (0, no pain, to 100, maximum pain) at baseline and 3 months postoperatively. The VAS is a widely used, reliable, and valid tool for measuring pain intensity with a minimally important change (MIC) of 28 points for the mean VAS pain, 32 points for VAS pain during physical load, and 27 points for VAS pain at rest in patients with DQ.^18,19^ The questionnaire asks patients to report the pain they experience in their affected hand. Also, hand function was scored with the patient-reported VAS score (0, no function, to 100, maximum function). The MIC for VAS function score in patients with DQ is 24 points.^19^ Patients’ medical records were retrospectively screened to identify comorbidities, including diabetes, thyroid disease, and other hand conditions besides DQ.

### Study Size

Power analysis for a hierarchical regression model including 23 predictors with a power of 0.80 and a significance level of 0.05, to detect a medium effect size defined by Cohen of 0.25, resulted in a required sample size of 529 patients.^20^ The current study exceeded this required sample size with a sample of 707 included patients.

### Statistical Analysis

The patient-reported outcome of surgery was analyzed in terms of the VAS pain score with a 0 to 100 scale. A paired *t* test was used to assess whether differences in VAS scores between baseline and 3 months postoperatively were significant. In addition, we used a hierarchical multivariable linear regression model to investigate the contribution of sociodemographic factors, disease-specific factors, and baseline pain and function scores to the VAS pain score 3 months postoperatively. The variables were added to the model in steps. We agreed upon the variables we have included based on previous knowledge in the literature and of our consulting experts before running the analysis. The first step involved sociodemographic factors, the second step disease-specific factors, and in the third and fourth step, baseline VAS pain and function scores were considered in association with postoperative VAS scores. After each step, the explained variance (r^2^) was calculated. The difference in r^2^ between steps is the relative contribution of the set variables to the change in the VAS score. Results are expressed in regression coefficients (B), representing the increase in the dependent variable for 1-unit increase in the independent variable when all variables remain constant. Standardized regression coefficients (β) were calculated to compare the relative contribution of each explanatory variable to the dependent variable. All model assumptions regarding normality, homoscedasticity, and multicollinearity were checked for possible biases. A *P* value of 0.05 or lower was considered statistically significant. All analyses were performed using R, version 4.0.5.^21^

## RESULTS

### Study Population and Baseline Characteristics

**Fig. 1.**
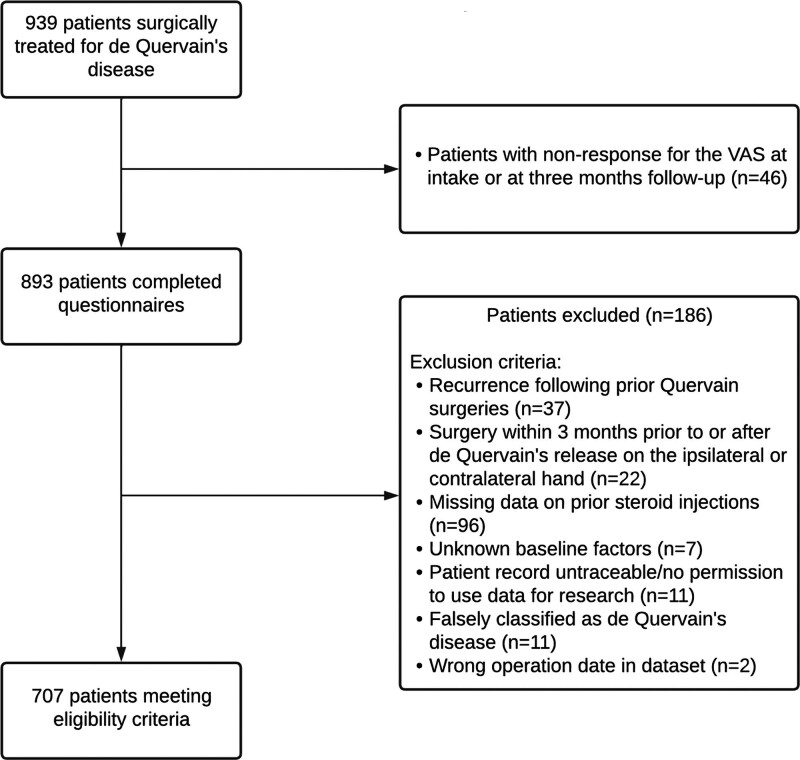
Flow diagram of the patients included in the study.

Between December of 2011 and September of 2019, 914 patients underwent a surgical release of the first extensor compartment. Of these patients, 46 did not respond to the VAS questionnaire (response rate, 95%). After applying the other eligibility criteria, 707 patients were included (Fig. 1). This cohort of 707 patients comprised 86% women, with a mean age of 52 years. Before surgery, the median symptom duration was 9 months (interquartile range, 6 to 12 months). Other demographic characteristics of the included patients are shown in Table 1. There was a significant difference only in BMI at baseline between included and excluded patients. [**See Appendix, Supplemental Digital Content 1**, which shows a nonresponder analysis comparing baseline characteristics of patients who did (*responder*) or did not (*nonresponder*) complete the VAS questionnaire at baseline or 3-month follow-up. Significance testing was performed using the independent-sample *t* test, http://links.lww.com/PRS/G637.]

**Table 1.** Baseline Characteristics of the Study Sample (*n* = 707)

Variables	Values
Patient characteristics	
Mean age (SD), yr	52 (13)
Female sex, %	86
Mean BMI (SD)	27 (5)
Current smoking, %	19
Former smoking, %	34
Dominant hand affected, %	58
Comorbidities, %	
Diabetes	8
Thyroid disease	8
Trigger finger	5
Trigger thumb	4
Carpal tunnel syndrome	16
Dupuytren disease	1
CMC1 osteoarthritis	11
CMC1 instability	5
History of hand trauma	11
Type of work, %	
Not employed (eg, unemployed or retired)	31
Light physical labor (eg, working in an office)	25
Moderate physical labor (eg, working in a shop)	32
Heavy physical labor (eg, construction work)	12
Clinical characteristics	
Median duration of symptoms (IQR), mo	9 (6, 12)
Concomitant surgery, %	8
Previous treatment with steroid injection, %	83
Baseline VAS scores (range, 0 to 100)	
Mean VAS pain (SD)	63 (19)
Mean VAS hand function (SD)	44 (23)

CMC1, carpometacarpal.

### Postoperative Pain and Hand Function

**Fig. 2.**
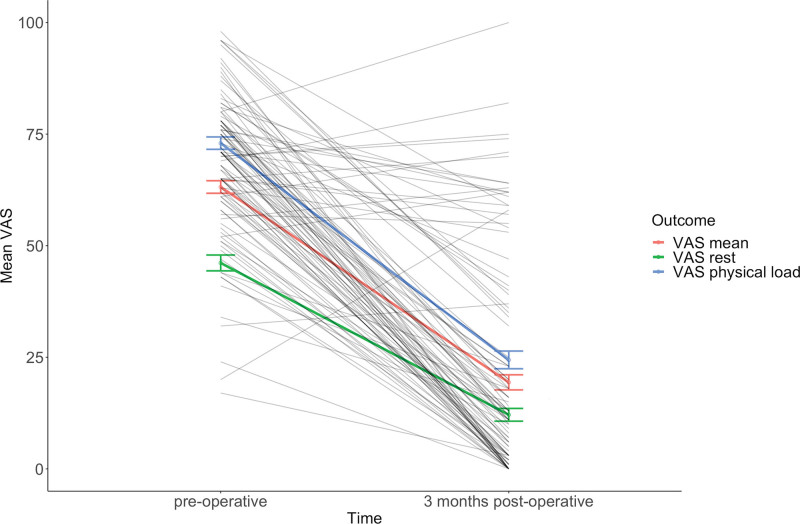
Postoperative course of the mean visual analogue scale (*VAS*) pain score, VAS pain score at rest, and VAS pain score during physical load. VAS scores range from 0 to 100, in which higher scores represent more pain. The error bars represent the standard error of the mean. All three categories of pain scores decreased over time. In the background, the mean VAS pain scores of 100 random patients are presented to depict the variation among patients. Some patients still reported pain postoperatively.

All VAS domains improved significantly after surgical release. On average, mean VAS pain decreased by 44 points (95% CI, 42, 46), from 63 at baseline to 19, 3 months postoperatively (*P* < 0.01) (Fig. 2). VAS pain during physical load decreased with a mean difference of 49 points (95% CI, 32, 36), and at rest with a mean difference of 34 points (95% CI, 31, 36) (*P* < 0.01) (Table 2). Furthermore, compared with baseline, the VAS function score improved with a mean difference of 34 points (95% CI, 31, 36), from 44 to 78, 3 months postoperatively (*P* < 0.01). Despite the decrease in pain, we found variability in individual outcomes; the proportion of patients reporting pain (mean VAS pain above 30) following surgery was 23% (*n* = 169). Seven percent (*n* = 51) of the patients reported a higher postoperative VAS pain score compared with the preoperative pain score.

**Table 2.** Mean VAS Pain and Function Outcomes^a^

Mean VAS Scores	Mean ± SD	Mean Difference	95% CI	*P*
VAS pain				
At intake	63.1 ± 19.1	43.7	41.8 to 45.8	<0.001
At 3 months	19.4 ± 22.9			
VAS rest				
At intake	46.1 ± 24.1	34.0	32.0 to 36.0	<0.001
At 3 months	12.1 ± 19.4			
VAS physical load				
At intake	73.0 ± 18.8	48.6	46.3 to 50.8	<0.001
At 3 months	24.4 ± 26.8			
VAS function				
At intake	44.2 ± 23.0	−33.7	−36.2 to −31.3	<0.001
At 3 months	77.8 ± 26.4			

a VAS pain score range: 0 (no pain) to 100 (maximum pain); VAS function score range: 0 (no function) to 100 (maximum function). Significance testing for the mean difference in VAS score at 3 months was performed using a paired *t* test with two groups: the preoperative VAS score at intake and the postoperative VAS score at 3 months.

### Factors Associated with Pain at 3 Months Postoperatively

The hierarchical regression model demonstrated that smoking (B = 6.37; 95% CI, 1.60, 11.15), a longer duration of complaints (B = 0.13; 95% CI, 0.04, 0.22), combined surgery (B = 14.40; 95% CI, 6.67, 22.13), and higher baseline VAS pain scores (B = 0.15; 95% CI, 0.06, 0.23) were associated with worse patient-reported pain at 3-month follow-up, when adjusting each factor for patient characteristics, disease-specific characteristics, and VAS intake scores. In addition, higher age was associated with lower pain intensity postoperatively (B = −0.35; 95% CI, −0.50, −0.19). The standardized regression coefficients (β) of all significant variables showed that combined surgery (β = 0.63) and smoking (β = 0.28) were the most influential factors. The final hierarchical regression model is presented in Table 3.

**Table 3.** Multivariable/Hierarchical Linear Regression Model for Mean VAS Pain Score at 3-Month Follow-Up^a^

Variables	B	95% CI	β	*P*
Sociodemographic factors				
Age, yrs	−0.35	−0.50 to −0.19	−0.20	<0.001^b^
Sex (men)	−1.41	−6.10 to 3.29	−0.06	0.557
BMI	0.14	−0.21 to 0.49	0.03	0.441
Smoking (yes)	6.37	1.60 to 11.15	0.28	0.009^b^
Former smoking (yes)	2.60	−1.21 to 6.41	0.11	0.181
Comorbidities (yes)				
Diabetes	−0.69	−7.38 to 5.99	−0.03	0.839
Thyroid disease	−3.85	−9.22 to 1.53	−0.17	0.160
Trigger finger	−2.69	−10.08 to 4.70	−0.12	0.475
Trigger thumb	2.76	−7.03 to 12.55	0.12	0.580
Carpal tunnel syndrome	−1.36	−5.97 to 3.26	−0.06	0.564
Dupuytren disease	5.50	−14.04 to 25.03	0.24	0.581
CMC1 osteoarthritis	5.36	−0.76 to 11.47	0.23	0.086
CMC1 instability	−0.43	−8.29 to 7.44	−0.02	0.915
Trauma	4.80	−1.04 to 10.64	0.21	0.107
Workload				
Unemployed (reference)				
Light physical work	−3.16	−7.60 to 1.29	−0.14	0.163
Moderate physical work	−3.20	−7.68 to 1.28	−0.14	0.161
Heavy physical work	0.14	−5.90 to 6.18	0.01	0.963
Disease-specific factors				
Dominant hand affected (yes)	2.22	−1.16 to 5.59	0.10	0.197
Duration of complaints, mo	0.13	0.04 to 0.22	0.10	0.004^b^
Combined surgery (yes)	14.40	6.67 to 22.13	0.63	<0.001^b^
Preoperative injections (yes)	2.51	−1.87 to 6.89	0.11	0.261
VAS				
VAS pain at intake	0.15	0.06 to 0.23	0.12	<0.001^b^
VAS function at intake	−0.06	−0.14 to 0.01	−0.06	0.090
Multiple R^2^	0.14			
Adjusted R^2^	0.11			

CMC, carpometacarpal.

a Results are presented as regression coefficients (B) with corresponding 95% CIs and standardized regression coefficients (β).

b Statistically significant (*P* < 0.05).

The final model explained the 11% variance in postoperative pain at 3 months of follow-up. After analyzing the contribution of the different groups of variables, we found that patient characteristics (eg, age, sex, and BMI) explained 5% of the variance in the mean VAS pain score at 3-month follow-up. Disease-specific characteristics (eg, duration of complaints, combined surgery, preoperative steroid injections) explained a further 4% variance in the postoperative mean VAS pain score. After adding the baseline VAS pain and, as the last step, baseline VAS function scores, the explained variance increased by another 2%, to a total of 11%.

## DISCUSSION

Surgical release for DQ is known to have a beneficial effect on pain. However, individual outcomes are variable. Therefore, it is valuable to examine whether preoperative clinical factors serve as prognostic factors for the outcomes of surgical treatment for DQ. This study confirms that surgical release significantly improved pain scores, and the benefit exceeded the MIC. However, a small group of patients experienced residual pain after surgery (23%). Several patient factors were found to entail worse pain scores at 3 months postoperatively (ie, younger age, smoking, longer duration of complaints, concomitant surgery, higher VAS pain scores at intake). This indicates that concomitant surgery does not always serve patients’ needs and might need to be reconsidered in some patients.

Of all baseline characteristics, smoking was among the most influential factor for higher patient-reported pain scores at 3-month follow-up, with a β of 6.37. This indicates that patients who smoke compared with patients who do not smoke score, on average, 6 points higher on a 0 to 100 VAS pain scale. This association between smoking and worse patient-reported outcomes after surgery for DQ aligns with earlier study results. In a retrospective study, Bryant et al.^14^ demonstrated that tobacco use is an independent risk factor for complications after surgical release, with an odds ratio of 2.31 (95% CI, 1.41, 3.79). In addition, incurring complications is associated with higher reported pain scores postoperatively.^22^

The lack of a non-significant association between comorbidities and VAS pain score 3 months after surgery was unanticipated. The same study by Bryant et al.^14^ described a negative association between diabetes and complications after surgery for DQ, with an odds ratio of 2.45 (95% CI, 1.51, 3.96). Bakhach et al.^23^ suggested that concurrent carpal tunnel syndrome influenced the outcome after surgery for DQ. Moreover, patients with carpometacarpal osteoarthritis were expected to demonstrate higher patient-reported pain scores 3 months after surgery, because DQ and carpometacarpal osteoarthritis frequently coexist.^24,25^ However, our results did not confirm an association between comorbidities and patient-reported postoperative pain. An explanation could be that only patients with an American Society of Anesthesiologists physical status of 1 or 2 are operated on in the participating clinics. However, when patients had another hand condition surgically treated simultaneously with the surgical release for DQ, the comorbidity interfered with higher patient-reported pain scores after surgery, with a β of 14.40. The β indicates that, on average, patients undergoing combined surgery score 14 points higher on the VAS pain scale (range, 0 to 100) postoperatively. This may explain why the results of Bakhach et al.^23^ and Bryant et al.^14^ differ from our results. They included cases in which other hand conditions were diagnosed or treated simultaneously with the surgery for DQ.^14,23^ Other procedures performed simultaneously with the first extensor compartment release in this study are summarized in Table 4. Among the 54 combined procedures, two patients developed complications from the other procedure. One patient who received a carpal tunnel release developed a wound infection, and one who received a ganglion extirpation had a recurrent ganglion. However, those complications did not affect the outcomes at 3-month follow-up.

**Table 4.** Overview of Other Procedures Performed Simultaneously with the First Extensor Compartment Release for de Quervain Disease^a^

Type of Surgery	No. (%)
Ganglion extirpation	20 (3)
Trigger finger release	16 (2)
Carpal tunnel release	7 (1)
Superficial radial nerve neurolysis	1 (0,1)
Dolphin tenotomy	1 (0,1)
Intersection syndrome release	2 (0.3)
Percutaneous needle fasciotomy	2 (0.3)
Synovectomy	5 (0.7)

a Values are reported as the number of cases with a percentage computed over the entire study population (*n* = 707).

Another relevant finding was that when patients experienced symptoms for a longer period before surgical release, they had worse outcomes in terms of postoperative pain. Our results show that for each additional year a patient experienced symptoms before surgery, they scored on average 1.6 points higher on the VAS pain scale at 3-month follow-up. Clinically, this effect is small; therefore, it might be questioned whether we should operate on patients with DQ earlier instead of continuing with conservative treatment if this does not improve pain. The multidisciplinary international treatment guideline based on expert opinions written by Huisstede et al.^26^ described symptom duration as one of the main factors in choosing a treatment option for DQ. In this guideline, the authors used a cutoff value of 6 months, at which point immediate surgery is required because of substantial symptoms. However, in the literature, there are no explanations for why patients with a longer symptom duration have worse patient-reported pain scores after surgery. One explanation might be that patients with chronic DQ symptoms have more tenosynovitis and therefore need longer recovery times. Another explanation could be that the superficial radial nerve will be affected because of a longer duration of symptoms. Nerve repair requires a longer recovery time, and perhaps these patients would experience improvement after a longer follow-up period.^27^

No association was found between receiving preoperative corticosteroid injections and outcome after surgery. For trigger finger, which is also a tenosynovitis, Koopman et al.^16^ identified factors associated with self-reported pain after surgical A1 pulley release. They found that receiving corticosteroid injections before surgery was associated with worse postoperative pain scores, especially when receiving three or more injections.^16^ The current study did not assess the relevance of the number of injections administered preoperatively, although our study demonstrated that corticosteroid injections (irrespective of the number of injections) before surgery for DQ do not affect the postoperative outcome. Previous literature has not examined this correlation for DQ. However, the previously mentioned treatment guideline recommends that only a limited number of corticosteroid injections should be administered preoperatively, with a maximum limit of three injections.^26^ Because it has been demonstrated that receiving three or more injections was associated with worse postoperative pain scores for trigger finger, it is possible that the exact number of injections before surgery also matters for the surgical treatment outcome of DQ. Further research is required to determine this relationship. However, based on our results, corticosteroid injections generally do not affect the surgical outcome after failed conservative treatment and could therefore be considered as conservative therapy for DQ.

**Fig. 3.**
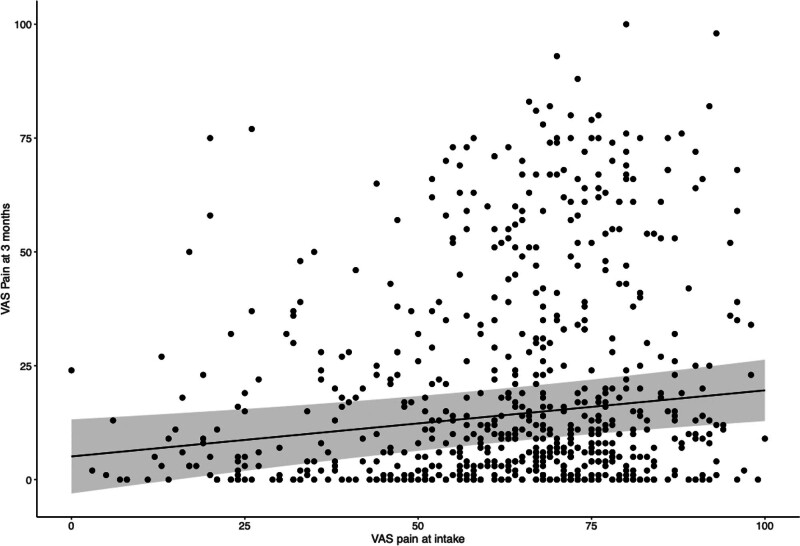
Association between VAS score for pain at 3 months and VAS at intake. A higher VAS intake score was associated with a worse VAS score at 3-month follow-up. The *points* represent individual patients. The *black* line is the linear relationship; the *shaded areas* mark the upper and lower boundary of the 95% CI.

Other variables affecting the VAS pain score at 3-month follow-up were age and a higher VAS pain intake score (Fig. 3). Age had a β of −0.35 per year in our model. This indicates that for each year patients are younger, 0.35 points are added to the VAS pain score. For example, a 22-year-old patient with DQ will, on average, score 10.5 points higher on a VAS scale at 3-month follow-up compared with an otherwise similar 50-year-old patient with DQ. In addition, the VAS pain at intake score had a β of 0.15 in our model. This implies that for each point patients report more pain on intake, the VAS pain score increases by 0.15 points at 3-month follow-up. For example, patients with a VAS pain score of 80 points at intake score, on average, nine points higher on a VAS pain scale than patients with an intake score of 20 points. Although some of these effects seem small, several characteristics often coexist in patients, substantially affecting the surgical outcome. For example, patients with an unfavorable profile (young patients who smoke, have complaints for 12 months, and have a high VAS pain intake score of 80 points) report a considerably higher average pain intensity (29 points difference) compared with patients with a favorable profile.

Our final model explained 11% of the variance in postoperative patient-reported pain. This suggests that the majority of the variation in pain for different patients cannot be explained by variables included in this study. It remains difficult to predict which patients will worsen or improve after surgery, despite the comprehensiveness of the clinical variables we included in our model. In a study of 164 patients surgically treated for DQ, a multivariable model showed that psychologic factors, such as patient expectations before surgery and illness perception, explained 31% of variance.^11^ However, that study considered other outcome measures—such as the Patient-Rated Wrist/Hand Evaluation—therefore, this variable cannot be directly compared. An explanation for this could be that the Patient-Rated Wrist/Hand Evaluation score focuses more on hand function, whereas the VAS questionnaire used in this study focuses more on pain, because patients with DQ experience more pain rather than a restriction in function.

A strength of our study is the size of the study population. We were able to test our hypothesis with a large number of completed VAS questionnaires because of the unique registration system on clinical outcomes. As a result, there was enough power to detect a true result with small confidence intervals. In addition, 95% of our cohort completed all questionnaires, even though participation was voluntary. The nonresponder analysis only showed a difference in BMI between patients who completed questionnaires and patients who did not (**see Appendix, Supplemental Digital Content 1**, http://links.lww.com/PRS/G637).

Several limitations should also be considered. First, the number of comorbidities might have been underestimated because of the retrospective search in patients’ medical records. Furthermore, some comorbidities might have been missed by physicians during consultation. However, other variables were obtained prospectively. Second, our model could not assess information on comorbidities, such as centralized pain conditions (eg, fibromyalgia) or nerve disorders (eg, superficial radial nerve neuropathy), because of inconsistent registration. Another relevant factor missing in our analysis is the preoperative use of narcotics. Because our outcome measure consisted of patient-reported pain scores, this factor may affect our results, as reported by Kazmers et al.^28^ for patient-reported functional and psychologic outcomes after hand surgery. None of the patients used neuropathic pain medication. However, over-the-counter antalgic medication was not registered during regular care. The same applies to assessing a correlation between a subcompartment and residual pain after a surgical release. In 40% of the population, a subcompartment separating the extensor pollicis brevis and abductor pollicis longus is identified.^29^ However, because all patients in our cohort were checked for a subcompartment during surgery, and, if necessary, this compartment was released, we do not expect residual pain caused by an incomplete release of a subcompartment. Moreover, we did not assess the type of incision, because our meta-analyses showed that this does not affect the outcome following surgery.^8^ In addition, recall bias should be considered concerning the variable duration of complaints. This limitation may be partially mitigated as patients fill out this variable together with their hand therapist in their first consultation. Finally, we looked at patient-reported outcomes at 3-month follow-up. However, previous literature has followed patients over an extended period. Kang et al.^30^ showed that between 12- and 24-month follow-up, there was continuing improvement. Therefore, it is possible that our patients also improved after our last measurement.

## CONCLUSIONS

This study confirms that surgical release in patients with DQ significantly reduces patient-reported pain 3 months after surgery. Smoking, concomitant surgery, and higher VAS pain scores at intake are associated with worse patient-reported pain at 3-month follow-up. A longer duration of complaints is also an influential factor for higher reported pain. While this confirms current guidelines advising not to delay conversion to surgery for too long, the effect of duration of complaints is small, suggesting that it should not be considered the only important factor.

## ACKNOWLEDGMENT

The authors thank the patients who participated and allowed their data to be used anonymously for the study.

## DISCLOSURE

The authors have no financial interests to disclose. None of the authors has a potential conflict of interest concerning this article’s research, authorship, or publication. No financial support was received for this work.

## Supplementary Material



## APPENDIX

The collaborators of the Hand-Wrist Study Group are as follows: R.A.M. Blomme, A. Kroeze, J. Smit, J. Debeij, K. Harmsen, B.J.R. Sluijter, D.J.J.C. van der Avoort, G.M. van Couwelaar, G.J. Halbesma, R. Koch, G.M. Vermeulen, J.P. de Schipper, J.F.M. Temming, J.H. van Uchelen, H.L. de Boer, K.P. de Haas, O.T. Zöphel, R. Feitz, J.S. Souer, S.E.R. Hovius, T.M. Moojen, X. Smit, C. van Nieuwenhoven, B. van der Heijden, J.W. Colaris, R. van Huis, P.Y. Pennehouat, K. Schoneveld, Y.E. van Kooij, R.M. Wouters, J. Veltkamp, A. Fink, W.A. de Ridder, H.P. Slijper, R.W. Selles, R. Poelstra, M.J.W. van der Oest, L. Hoogendam, J.S. Teunissen, J.E. Koopman, J. Dekker, W.R. Bijlsma, and M.H.P. ter Stege.
