# Supplementary material for: Preoperative Indicators of the Effectiveness of Surgical Release in Patients with de Quervain Disease: A Prospective Cohort Study
Source: Plast Reconstr Surg. 2023 Nov 23;153(5):952–61. doi: 10.1097/PRS.0000000000010445 (PMC11027984; doi:10.1097/PRS.0000000000010445)
Supplement: Supplementary file 1 [file prs-153-952e-s001.pdf]

Supplementary material

Appendix 1: Non-responder analysis comparing baseline characteristics of patients who did (responder) and who did not (non-responder) complete the Visual Analog Scale (VAS) questionnaire at baseline or three months follow-up. Significance testing was performed using the independent sample t-test.

Variable	Responder (n = 893)	Non-responder (n = 46)	P-value
**Patient characteristics**			
Age in years, mean (SD)	52 (13)	55 (13)	0.14
Female sex, %	85%	87%	0.95
Body Mass Index (BMI), mean (SD)	27 (5)	25 (3)	0.02
Current smoking, %	19%	20%	0.95
Former smoking, %	33%	33%	
Comorbidities, %			
Diabetes	7%	11%	0.54
Thyroid disease	9%	9%	1.00
Trigger finger	6%	4%	0.90
Trigger thumb	4%	2%	0.71
Carpal Tunnel syndrome	17%	15%	0.91
Dupuytren's disease	1%	2%	1.00
CMC1 osteoarthritis	11%	13%	0.79
CMC1 instability	4%	4%	1.00
History of hand trauma	10%	9%	0.90
Type of work, %			0.808
Not employed (e.g., unemployed or retired)	31%	26%	
Light physical labour (e.g., working in an office)	25%	22%	
Moderate physical labour (e.g., working in a shop)	32%	37%	
Heavy physical labour (e.g., construction work)	13%	11%	
**Clinical characteristics**			
Dominant hand affected, %	56%	54%	0.98
Duration of symptoms in months, median (IQR)	8 [5, 12]	8 [5, 12]	0.44
Concomitant surgery, %	9%	2%	0.20
Pre-operative steroid injections, %	69%	54%	0.48
**Baseline VAS scores (range 0-100)**			
VAS Pain, mean (SD)	64 (19)	65 (20)	0.74
VAS Hand function, mean (SD)	43 (23)	50 (31)	0.17

Abbreviations: SD, Standard deviation; IQR: Interquartile range; CMC: Carpometacarpal; VAS, Visual Analog Scale.
